# Influence of Hyperglycaemia and CRP on the Need for Mechanical Ventilation in Guillain-Barré Syndrome

**DOI:** 10.3389/fneur.2022.875714

**Published:** 2022-05-23

**Authors:** Ivana Štětkářová, Edvard Ehler, Michal Židó, David Lauer, Jan Polák, Jiří Keller, Tomáš Peisker

**Affiliations:** ^1^Department of Neurology, Third Faculty of Medicine, Královské Vinohrady University Hospital, Prague, Czechia; ^2^Department of Neurology, Faculty of Health Studies, Pardubice University and Pardubice Regional Hospital, Pardubice, Czechia; ^3^Department of Pathophysiology, Third Faculty of Medicine, Charles University, Prague, Czechia; ^4^Department of Radiology, Na Homolce Hospital, Prague, Czechia

**Keywords:** Guillain-Barré syndrome, mechanical ventilation, C-reactive protein, hyperglycaemia, CRP

## Abstract

**Objectives:**

Elevated blood glucose and CRP (C-reactive protein) are usually related to a worsened clinical outcome in neurological diseases. This association in Guillain-Barré syndrome (GBS) has been studied rarely. We tried to analyse if hyperglycaemia and CRP at admission may influence the outcome of GBS, including mechanically ventilated (MV) patients.

**Methods:**

We retrospectively studied 66 patients (40 males, 19–93 years, average 56 years) without diabetes mellitus and free of corticoid treatment, who fulfilled the clinical criteria for diagnosis of GBS. Hyperglycaemia (the level of fasting plasma glucose, FPG) was defined as blood glucose level >5.59 mmol/L according to our laboratory. CRP >5 mg/L was considered as an abnormally elevated value.

**Results:**

At admission, 32 GBS patients (48%) had hyperglycaemia according to FPG level. A severe form of GBS (>4 according to Hughes GBS scale) was observed in 17 patients (26%); and 8 of them (47%) had hyperglycaemia. Fourteen patients (21%) were MV, and in 10 of them (71%) hyperglycaemia was present. CRP was significantly increased in MV patients. The linear model revealed a significant relationship between CRP and glycemia (*p* = 0.007) in subjects without MV (*p* = 0.049). In subjects with MV the relationship was not significant (*p* = 0.2162, NS).

**Conclusion:**

In the acute phase of GBS at admission, hyperglycaemia and higher CRP occur relatively frequently, and may be a risk factor for the severity of GBS. Stress hyperglycaemia due to impaired glucose homeostasis could be one explanation for this condition.

## Introduction

Guillain-Barré syndrome (GBS) is an autoimmune inflammatory disease of the peripheral nervous system. The incidence of GBS is 1–2/100,000 inhabitants per year (1, 2). GBS is more common in men than in women and increases with age, although it may occur in lower age groups (3).

Typical clinical signs of GBS include weakness and numbness in the lower limbs, which has an increasing tendency and progresses to the upper limbs (3, 4). The diagnosis of GBS is based on a history of previous infectious diseases, especially of the respiratory and gastrointestinal tract, a clinical picture including ascending motor weakness with sensory impairment, neurophysiological findings documented on EMG and examination of cerebrospinal fluid (1, 5–7). Neurophysiological examination will determine the individual subtypes of GBS—acute inflammatory demyelinating polyradiculoneuropathy (AIDP), acute motor axonal neuropathy (AMAN) and acute motor and sensory axonal neuropathy (AMSAN), and other rare GBS variants.

The disease usually progresses rapidly, with the maximal peak of disability 2 weeks from the onset of the disease. About 20–30% of patients are placed on lung ventilation for severe respiratory muscle weakness (4, 8). After a certain time (weeks, up to months) the clinical condition slowly improves. About 60–80% of patients are able walk independently after 6 months from the onset of the disease, with or without treatment (9, 10).

Elevated blood glucose is common in different neurological diseases such as stroke, usually with worsening of the clinical outcome (11–13). The dysfunction of the immune response is critical in the pathogenesis of GBS as well as in diabetes mellitus including cytokines and antibodies to gangliosides (14, 15). An association between Guillain-Barré syndrome (GBS) and blood glucose level has been studied very rarely (16, 17). There are also very few studies in the literature that address the issue of predictive value of C-reactive protein in the early phase of GBS (18, 19). The level of CRP in peripheral blood has been reported to be significantly higher in GBS patients than in controls (20). Ning et al. have been recommended to use a novel inflammatory biomarker named CAR (CRP-to-albumin ratio), which is independently associated with the occurrence of respiratory failure and poor short-term outcome in GBS patients.

In light of this, the aim of this study was to retrospectively analyse if fasting glucose level at admission may influence the severity of GBS, including mechanically ventilated patients. We also tried to find the clinical outcome in relation to the presence of inflammatory conditions measured by C-reactive protein. We analyzed data from the cohort of patients of two large hospitals including clinical, neurophysiological, and cerebrospinal fluid findings.

## Methods

In this study we retrospectively studied 73 adult patients from the years 2012 until 2020, who fulfilled the clinical criteria for diagnosis of GBS and were admitted to the Department of Neurology, Charles University and Faculty Hospital Královské Vinohrady (25 cases) and Department of Neurology, Pardubice University and Pardubice Regional Hospital (48 cases). This retrospective study was approved by the Ethics Committee (EK-R/03/0/2020); though written informed consent was not obtained, patient information was strictly anonymised.

We excluded patients with Miller Fisher syndrome (one patient). Seven patients (9.6%) suffering from diabetes mellitus were also excluded and the final analysis was proceeded in 66 patients (40 males, 19–93 years, average 56 years) free of corticoid treatment.

Hyperglycaemia (the level of fasting plasma glucose, FPG) was defined as blood glucose level >5.59 mmol/L according to our laboratory (normal laboratory reference value of serum glucose ranged from 3.90 to 5.59 mmol/L). After admission, blood tests were performed on fasting patients in the mornings (6:00–6:30 a.m.). At the same time CRP was performed in serum. In our hospitals, the normal laboratory reference value of serum CRP was defined as below 5 mg/L. Lumbar puncture was performed in all patients but one 1–2 weeks after the onset of the disease. Clinical assessment of motor functions used the GBS disability scale (21), ranging from 0 to 6 as follows: 0: healthy; 1: minor symptoms and capable of running; 2: able to walk 5 m or more without assistance but unable to run; 3: able to walk 5 m across an open space with help; 4: bedridden or chair-bound; 5: requiring assisted ventilation for at least part of the day; 6: dead.

Data from GBS patients were obtained through their history and chart review, including occurrence of pre-diagnosis events such as flu-like disorders, diarrhea, fever, operation or vaccination; severity of muscle weakness assessed by the GBS disability scale; cerebrospinal fluid (CSF) analysis including protein and cell level; CSF and serological assessment for antiganglioside antibodies against GM1, GM2, GD1a, GD1b, and GQ1b; EMG testing including nerve conduction studies on the upper and lower extremities divided GBS into demyelinating or axonal subtypes; treatment regime of intravenous immunoglobulin of 0.4 g/kg body weight daily for 5 days and plasma exchange of 200–250 ml plasma/kg body weight in five sessions; mechanically ventilated GBS patients. Statistical analysis was conducted in R (22).

## Results

Demographics, clinical symptoms, neurological findings, neurophysiology, CSF, and other laboratory findings are summarized in Table 1.

**Table 1:** Demographic, clinical, laboratory and treatment data in patients with Guillain-Barré syndrome.

		**Number**	**%**	**Range**
**Demographic data**				
	Male	40	61	
	Female	26	39	
	Age	55^a^		19–93
Pre-clinical manifestation				
	Upper respiratory tract infection	22	33	
	Diarrhea	11	17	
	Operation	4	6	
	Fever and muscle pain	3	5	
	Absent	26	39	
Clinical manifestation				
	Motor and sensory	52	79	
	Only motor	6	9	
	Only sensory	1	1	
	Cranial nerve involvement	7	11	
	Respiratory failure	14	21	
	Hughes GBS scale at admission 1–3	49	74	
	Hughes GBS scale at admission 4–6	17	26	
**Initial laboratory**				
Glycaemia in serum				3.54–6.84 mmol/L
	Normal	34	52	
	> 5.59 mmol/L	32	48	
CRP in serum				0.3–82.2 mg/L
	Normal	35	53	
	> 5 mg/L	31	47	
Gangliosides in serum^b^	Normal	38	63	
	Abnormal	22	37	
CSF protein level^c^				0.14–1.73 g/L
	Normal <0.44 g/L	17	26	
	> 0.44 g/L	48	74	
CSF cells/μl^c^				0–46^d^
	Normal <4 cells/μl	37	57	
	5–10 cells/μl	19		
	> 10 cells/μl	8^d^		
Electromyography				
Demyelinated form		60	91	
Axonal form		6	9	
Treatment				
Plasma exchange		32	48	
IVIG 2 g/kg		29	44	
No treatment		5	8	

a *Average*.

b *Six patients were not tested*.

c *One patient refused lumbar punction*.

d *One patient had 70 cells/μl and this number was not included; CRP, C-reactive protein; CSF, cerebrospinal fluid, IVIG, intravenous immunoglobulins*.

Clinical symptoms in our group included the classic sensorimotor form of GBS presented with distal paresthesia, accompanied by weakness that starts in the legs and progresses to the arms. We observed pure sensory symptoms and cranial nerves involvement (mostly facial nerve palsy) in only two patients. Reflexes were decreased or absent in most patients at admission to the hospital. Half of our patients report symptoms of a respiratory or gastrointestinal tract infection 1–2 weeks before the onset of GBS. Seventeen GBS patients had normal protein level in CSF > 0.44 g/L (23%), in the range of 0.14–1.73 g/L. All but one patient had a CSF cell count below 50 cells/μl. In 37% of GBS patients we detected positive findings of serum gangliosides.

Patients were treated by intravenous immunoglobulins of 0.4 g/kg daily for 5 days or plasma exchange of 200–250 ml/kg for 5 sessions.

Four patients died. All but one was mechanically ventilated as follows: 69-years-old female with stomach cancer; 78-years-old male with AH and chronic ischemic heart disease (CIHD), non-ventilated; 84-years-old male with AH and CIHD; 93-years-old female with heart failure and pneumonia.

At admission, 32 GBS patients (48%) had hyperglycaemia according to FPG level. A severe form of GBS (>4 according to Hughes GBS scale) was observed in 17 patients (26%); and 8 of them (47%) had hyperglycaemia. Fourteen patients (21%) were mechanically ventilated and in 10 of them (71%) hyperglycaemia was present. At admission, 32 GBS patients (48%) had a higher serum CRP level.

The FPG glycemia in subjects who required mechanical ventilation was higher than in those who were able to ventilate without support (mean 5.98 and 5.59, Wilcoxson single-tailed test *p*-value = 0.026). Comparison of binarized glycemia values (indicating increased glycaemia, values higher than threshold 5.59 mmol/L were taken as “1,” lower as “0”) resulted in a very similar *p*-value = 0.028. However, on admission CRP was higher in those who developed the need for a mechanical support as well (Wilcoxson single-tailed test *p*-value < 0.001).

**Figure 1:**
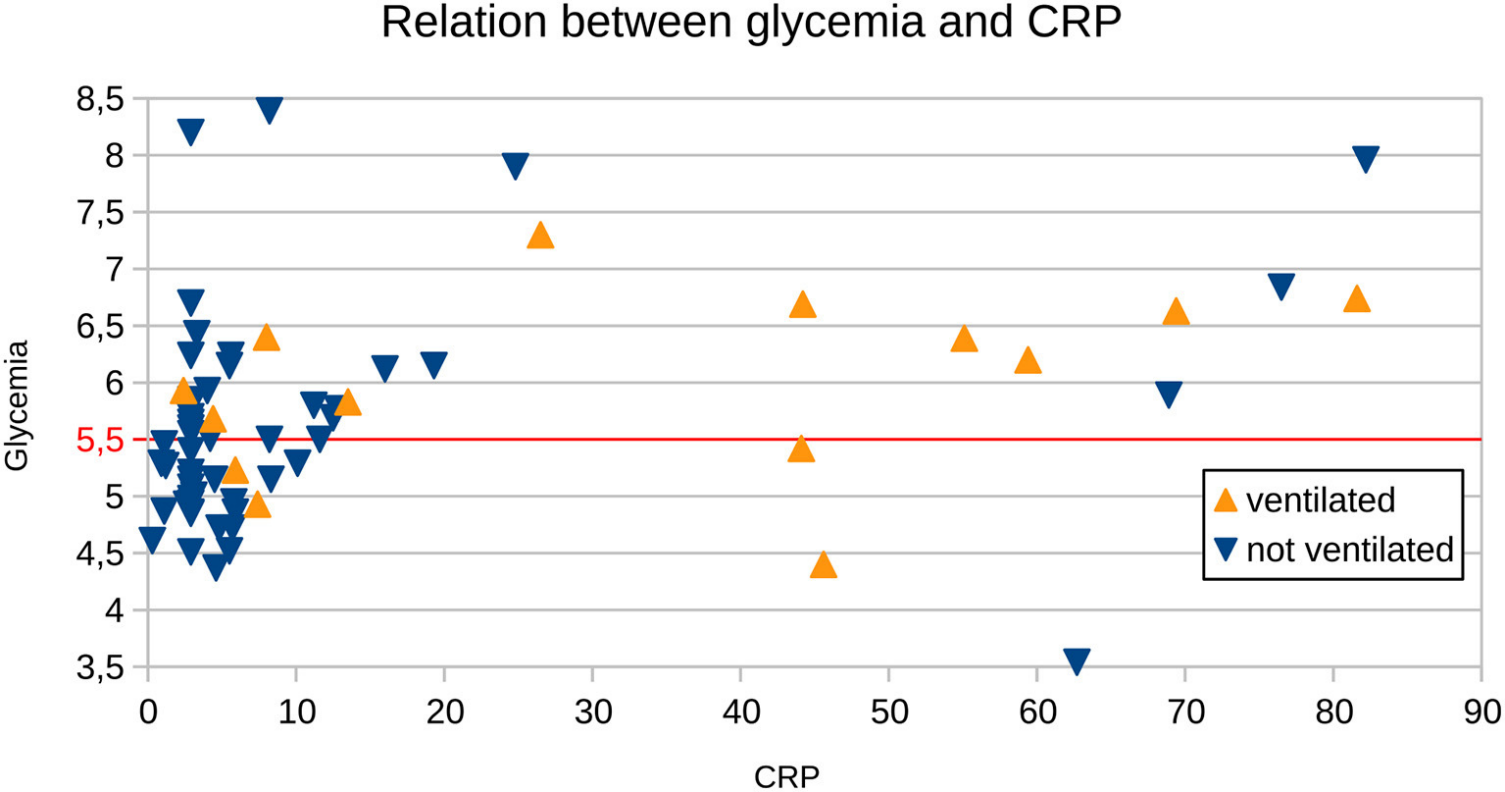
Relation between CRP and glycemia in subjects who did not require ventilation support (“not ventilated”) and those who did (“ventilated”). The red horizontal line represents a cut-off value for normal glycemia levels.

A linear model revealed a dependency between CRP and FPG glycemia level (*p* = 0.0075), which was present in subjects not requiring mechanical ventilation support (*p* = 0.0493) but not in ones for whom mechanical ventilation support was required (*p* = 0.2162—NS) (Figure 1).

## Discussion

Our retrospective study in GBS patients showed that FPG hyperglycaemia and increase of CRP at admission were positively associated with the need for mechanical ventilation. On the other hand, a low level of FPG glucose and normal value of CRP at admission may be useful parameters at the beginning that help to identify the patients in a mild course who do not reach such a severe clinical condition requiring mechanical ventilation. FPG hyperglycaemia at admission was found in more than half of the GBS patients in our cohort, but this parameter was not associated with a worse prognosis. One explanation may be that hyperglycaemia as a reflexion of stress-induced hepatic glucose output plays a protective role during the acute phase of the disease and it is an evolutionary adaptive response that increases the host's chances of survival (23). Even though the dependency between CRP and glycemia, found in our study in subjects who did not need mechanical ventilation support, does not have a direct clinical significance, we may speculate that in subjects with CRP over 25 mg/L further studies may find the aid for identification of the subjects who are going to be in need for the mechanical ventilation support, based on the laboratory results.

Guillain-Barré syndrome is an immune-mediated polyneuropathy, clinically characterized by symmetrical weakness of the limbs with hyporeflexia or areflexia, which reaches a maximum severity within 2–4 weeks from the onset. Two-thirds of patients report symptoms of a respiratory or gastrointestinal tract infection before the onset of GBS (24). In this study we found that half of our GBS patients had symptoms of such infection 1–3 weeks before the onset of the disease. Auto-antibodies to gangliosides appear in GBS more often (3). In this study mostly 37% of our GBS patients had positive serum gangliosides. In many studies, protein CSF levels were normal in 25–50% of patients, usually in the first week after disease onset (3, 4, 9). Here we observed normal protein CSF level in 26% of GBS patients, which is compatible with published data. Mild pleocytosis (10–50 cells/μl) presented in our cohort still prompted us to exclude other diagnoses, such as infectious causes.

Respiratory insufficiency requiring mechanical ventilation is observed in 20–25% of GBS patients (9, 24). In our cohort, a similar amount of GBS patients (21%) developed the need for pulmonary ventilation. Several models to predict the probability of respiratory insufficiency in the first week after admission for GBS were developed following the severity of muscle weakness, time interval between the onset of weakness and admission at the hospital, and facial and/or bulbar weakness (3, 4). In a recent study the authors followed inflammatory predictors such as CRP and CRP-to-albumin ratio for mechanical ventilation (16).

GBS is a serious disease with mortality variability between 3 and 7% (24, 25). Predictors of an increased risk of death are advanced age, severe disease, increased comorbidity, pulmonary and cardiac complications, mechanical ventilation, and systemic infection. In this study, four patients died (6%), all were in the higher age (range 69–93 years), with comorbidities and all but one was mechanically ventilated.

A normal result for fasting blood glucose ranges from 70 to 100 mg/dL (3.90 to 5.59 mmol/L). According to criteria set by the American Diabetes Association (ADA), higher than normal fasting blood sugar between 100 and 125 mg/dL (5.6 to 6.9 mmol/L) may indicate prediabetes. In our study, almost half of the people had higher glucose levels, which, according to the above ADA criteria, would already signal prediabetes. One of the explanations may be stress hyperglycaemia as a physiological adaptive mechanism to life-threatening insult. Higher fasting serum glucose levels ≥126 mg/dl (7.0 mmol/L) on the second day after admission was common in critically ill patients and appeared to be a marker of disease severity (23).

Wang et al. (16) retrospectively analyzed a large group of 350 GBS patients and found that hyperglycaemia at admission was positively associated with the severity of GBS at discharge. Moreover, a high level of serum glucose at the GBS onset related to severe motor weakness, cranial nerve involvement, autonomic involvement, and dyspnea. An association between hyperglycaemia and severity of GBS was explained by these authors as multi-factorial for example with higher levels of pro-inflammatory cytokines, increased stress and related hypercortisolism, release of epinephrine and other stress hormones, etc. (16). Other authors have reported that worse neurological status with disability at ICU discharge correlated with dysglycemia in mechanically ventilated Guillain-Barré syndrome patients (26). In this study, we found a similar observation that hyperglycaemia and increase of CRP at admission were positively associated with mechanical ventilation. On the other hand, it is worth mentioning that, in this study, of subjects with CRP higher than 30 (*n* = 11) only three had normal FPG glycemia (and of those only one required ventilation support) and there were seven subjects not requiring ventilation support and only four requiring it. Therefore, adding FPG hyperglycaemia may help to discriminate between these two groups, however our data is limited due to a small group size.

CRP is an acute-phase reactant protein synthesized in the liver in response to inflammation following activation of the complement system. This inflammatory reaction may be associated with GBS by activating the immune pathway. GBS, as a serious autoimmune disorder, may in these conditions induce a higher level of inflammation resulting in an increase in the serum C-reactive protein. CRP has already been used to predict the need for mechanical ventilation and prognosis in GBS patients with inconsistent results across studies (19, 27, 28). Altaweel et al. found in 24 GBS patients a statistically significant positive correlation between clinical severity assessed by the Hughes disability scale and serum CRP level (19). Recently, Ning et al. used the CRP-to-albumin ratio (CAR) as a prognostic score in 200 retrospectively analyzed GBS patients and found that CAR, a novel inflammatory biomarker, was independently associated with the occurrence of respiratory failure and poor short-term outcome (18). Some authors observed that, apart from higher CRP levels, higher age and elevated serum neutrophil/lymphocyte ratio also indicated worse GBS prognosis (27, 28). In other neurological diseases, in which the proportion of inflammation is presumed, for example in stroke, CRP is increased, and it has been associated with an unfavorable outcome (29).

## Conclusion

We conclude that CRP > 5 is a risk factor associated with the occurrence of respiratory failure in GBS patients and may predict worse GBS prognosis; however, FPG hyperglycaemia alone measured at admission is not connected with respiratory failure. Patients with GBS with less severe disease who did not require mechanical ventilation had significantly lower serum FPG glycemia and CRP levels than patients who were mechanically ventilated. Lower CRP can serve as a positive predictive marker associated with better prognosis.

Further studies are necessary with detailed understanding of these relationships to explore the underlying mechanisms. This may have broader applications in the fields of critical care neurology.

### Clinical/Research Implications and Future Directions

The current work has implications for clinical outcome of GBS patients, demonstrating how FPG hyperglycaemia and higher CRP at hospital admission could be a bad prognostic factor for mechanical ventilation. Future studies are needed to understand how the natural course of GBS develops at the beginning of the disease and if we can predict clinical outcome in severe GBS forms responding to pharmacological treatment such as correction of hyperglycaemia and/or inflammatory changes.

### Study Limitations

The relatively smaller sample size of our retrospective data limits our ability to make conclusions of these findings with regards to the broader GBS population. Future prospective studies involving a representative sample of GBS patients are needed to further validate inflammatory processes as well as hyperglycaemia as a predictive value for GBS patients at hospital admission.

## Data Availability Statement

The raw data supporting the conclusions of this article will be made available by the authors, without undue reservation.

## Ethics Statement

This study involving human participants was reviewed and approved by the Local Ethical Committee of our University Hospital (Ethical Committee of Královské Vinohrady University Hospital, No. EK-R/1/0/2020). Written informed consent for participation was not required for this study in accordance with the national legislation and the institutional requirements.

## Author Contributions

IŠ and EE had a major role in study conceptualization, design, interpretation of the findings, and writing of the manuscript. MŽ, DL, and TP provided data analyses. JK performed statistical analysis. JK and JP provided a critical review of the article. All authors contributed to the article and approved the submitted version.

## Funding

This research was supported by Ministry of Health of Czech Republic (DRO NHH, 00023884) and Charles University (Cooperatio Neuroscience and Neuroimaging, GAUK 120121).

## Conflict of Interest

The authors declare that the research was conducted in the absence of any commercial or financial relationships that could be construed as a potential conflict of interest.

## Publisher's Note

All claims expressed in this article are solely those of the authors and do not necessarily represent those of their affiliated organizations, or those of the publisher, the editors and the reviewers. Any product that may be evaluated in this article, or claim that may be made by its manufacturer, is not guaranteed or endorsed by the publisher.
